# Analysis of the diversity of tick-borne viruses at the border areas in Liaoning Province, China

**DOI:** 10.3389/fmicb.2023.1179156

**Published:** 2023-05-02

**Authors:** Yu Bai, Yang Li, Wenli Liu, Jing Li, Fengjuan Tian, Lei Liu, Xiaohu Han, Yigang Tong

**Affiliations:** ^1^Jiamusi University School of Basic Medicine, Jiamusi, China; ^2^College of Life Science and Technology, Beijing University of Chemical Technology, Beijing, China; ^3^Key Laboratory of Livestock Infectious Diseases, Ministry of Education, Shenyang Agricultural University, Shenyang, China

**Keywords:** Liaoning Province, ticks, virus diversity, tick-borne viruses, metagenomics

## Abstract

Ticks play a significant role in transmitting arboviruses, which pose a risk to human and animal health. The region of Liaoning Province, China, with abundant plant resources with multiple tick populations, has reported several tick-borne diseases. However, there remains a scarcity of research on the composition and evolution of the tick virome. In this study, we conducted the metagenomic analysis of 561 ticks in the border area of Liaoning Province in China and identified viruses related to known diseases in humans and animals, including severe fever with thrombocytopenia syndrome virus (SFTSV) and nairobi sheep disease virus (NSDV). Moreover, the groups of tick viruses were also closely related to the families of *Flaviviridae*, *Parvoviridae*, *Phenuiviridae*, and *Rhabdoviridae*. Notably, the Dabieshan tick virus (DBTV) of the family *Phenuiviridae* was prevalent in these ticks, with the minimum infection rate (MIR) of 9.09%, higher than previously reported in numerous provinces in China. In addition, sequences of tick-borne viruses of the family *Rhabdoviridae* have first been reported from the border area of Liaoning Province, China, after being described from Hubei Province, China. This research furthered the insight into pathogens carried by ticks in the northeastern border areas of China, offering epidemiological information for possible forthcoming outbreaks of infectious diseases. Meanwhile, we provided an essential reference for assessing the risk of tick bite infection in humans and animals, as well as for exploring into the evolution of the virus and the mechanisms of species transmission.

## Introduction

In recent decades, changes in the global natural ecology and frequent international trade have expanded the geographical distribution of tick species, while the number of potentials or known tick-borne pathogens continues to increase (Fang et al., 2015; Madison-Antenucci et al., 2020). Tick-borne viruses (TBVs) can trigger several serious zoonotic diseases: for example, severe fever with thrombocytopenia syndrome virus (SFTSV), a novel bunyavirus discovered in Henan Province of China in 2010, can cause fever, hemorrhage, and even death from multiple organ failure (Yu et al., 2011). Thus far, more than 20 provinces in China have reported the disease, with a national average mortality rate of 5.3%, and cases of SFTSV infection have successively been investigated in other Asian countries like Korea and Japan (Zhan et al., 2017; Li et al., 2021). Nairobi sheep disease virus (NSDV), which used to be endemic in eastern Africa, was first confirmed in 2015 in Liaoning Province, China, and accounts for fatal acute hemorrhagic gastroenteritis among small ruminants (e.g., sheep, and goats). NSDV infection in humans may produce symptoms with fever, headache, and vomiting (Yadav et al., 2011; Gong et al., 2015). Recently, numerous new tick-borne viruses have been observed globally that may be zoonotic, such as Jingmen tick virus (JMTV), Heartland virus (HRTV), Alongshan virus (ALSV), Songling virus (SGLV) and others (Qin et al., 2014; Shen et al., 2018; Wang et al., 2019; Ma et al., 2021). Further investigation of the tick virus genomic will be necessary.

Next-generation sequencing (NGS) and metagenomics analysis have demonstrated superiority in discovering novel viruses in ticks and various invertebrate vectors and exploring viral evolution. These viral sequences reveal evolutionary and genetic features of the genome, and some of them appear likely to be the phylogenetic ancestors of infected vertebrates. Not only does this reveal the diversity of viruses, but it also contributes to a greater extent to the understanding of the microbiome and thus to public awareness of the viral community (Li et al., 2015; Remnant et al., 2017; Pettersson et al., 2020; Cai et al., 2022). As the incidence of cross-species virus transmission increases, metagenomic sequencing (mNGS) has played a critically valuable role both in identifying novel pathogens and in gaining an appreciation of virus evolution, but also in providing innovative insights into virus-ecosystem interactions (French and Holmes, 2020). Jia et al. conducted the mNGS analysis of diverse tick species. The study demonstrated a correlation between the composition of the viral component of different tick species and ecological and geographical factors, which will emphasize the study of tick-borne viruses concerning the ecological factors regarding host species, life cycle, and geographical location. The outcomes will facilitate the characterization of the transmission of the virus and the assessment of its potential future threat (Jia et al., 2020; Xu et al., 2021).

Liaoning Province sits in northeastern China and belongs to a coastal city with a humid climate and abundant rainfall; it shares borders with North Korea, South Korea, and Japan, and serves as a key location for international trade. On the one hand, it possesses abundant forest and animal resources, which create favorable conditions for the growth and development of ticks. On the other hand, with urbanization and the expansion of human activity, the risk of tick bites and disease infection will increase. *Haemaphysalis longicornis* represents the dominant tick species in Liaoning Province, carrying more than 50 species of pathogens (Zhao L. et al., 2020). Consequently, there exists a necessity to strengthen the diagnosis and monitoring of tick-borne viruses to prevent the occurrence of serious epidemics. In this work, a metagenomic sequencing methodology has been employed to identify and classify the gene sequences of viruses carried by ticks in the border areas of Liaoning Province and to initiate surveillance of novel insect-borne pathogens. This not only provides a rapid understanding of the known and unknown viruses carried by ticks but also enriches the viral genome database, which is fundamental for the prevention and control of the spread of major and emerging epidemics.

## Materials and methods

### Sample collection

**Figure 1:**
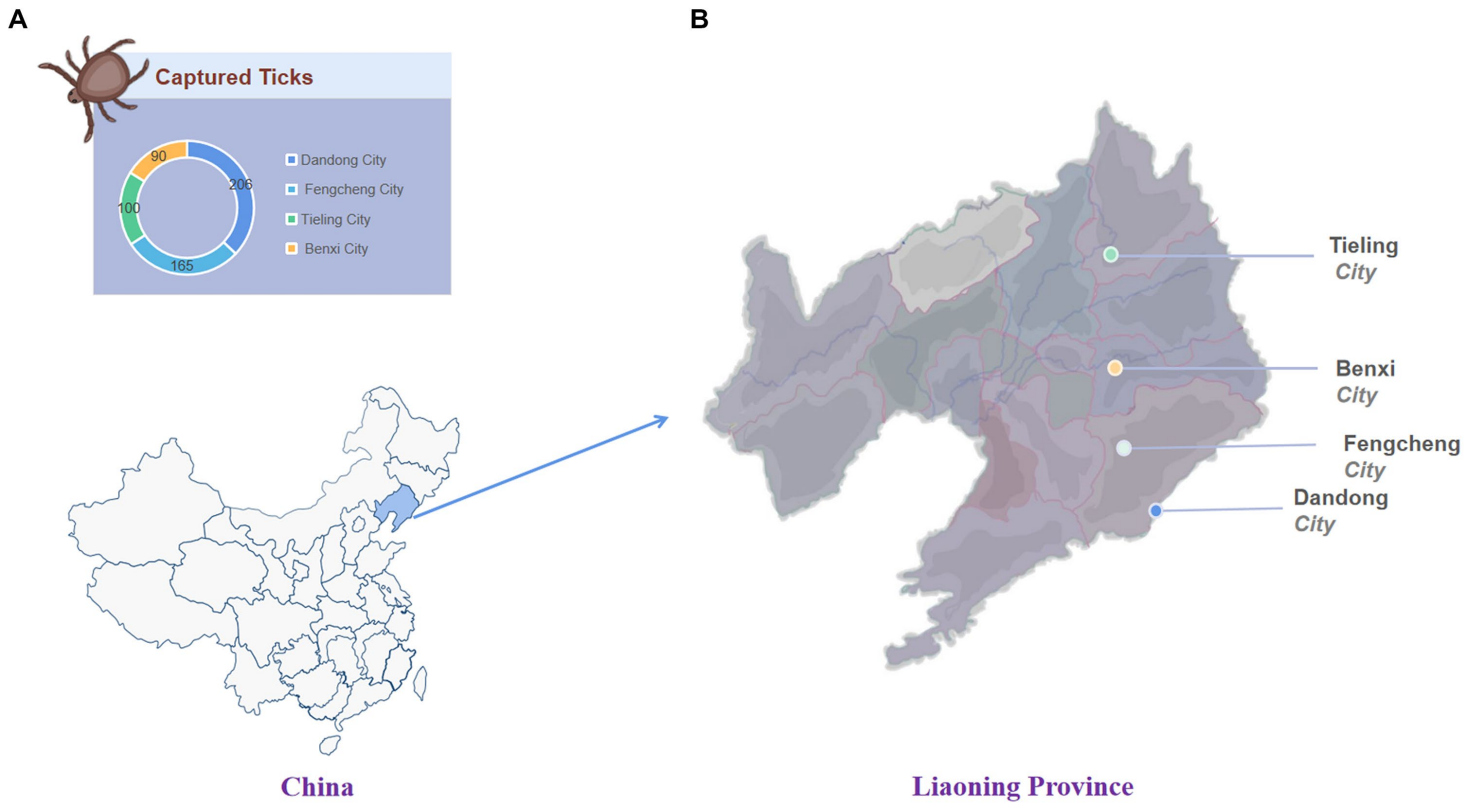
The number and location of ticks collected. **(A)** The number of ticks captured in each area. **(B)** Tick collection area in Liaoning Province.

From May to July 2022 (peak season for tick activity), a total of 561 ticks were collected using the drag-flag method from 14 sites in four border cities in eastern Liaoning Province, namely Dandong city (40°07′ N, 124°23′ E), Fengcheng city (40°45′ N, 124°06′ E), Tieling city (42°28′ N, 123°83′ E), and Benxi city (41°24′ N, 124°17′ E) (Figure 1 and Supplementary material S1). Ticks were initially identified morphologically based on entomology and further identified by sequencing data against the mitochondrial cytochrome c oxidase subunit I (COI) gene (Zhao T. et al., 2020). Ticks were transported to the laboratory in labeled test tubes on dry ice.

### Sample preparation and RNA extraction

According to the tick collection area, 561 ticks were divided into 58 groups, respectively, into 1 mL phosphate buffered saline (PBS), adding zirconium beads, and grinding in a frozen grinder at 60 hz for 10 min. After centrifugation at 12,000 × g for 5 min, 300 μL tick supernatant was absorbed into 0.45 nm filter membrane and centrifuged at 12,000 × g for 3 min to remove excess tick cells and bacteria as much as possible. Total viral nucleic acid was extracted using the OMEGA E.Z.N.A.^®^ SE Viral DNA/RNA Kit according to the manufacturer’s protocol by sieving 250 μL filtrate into a 1.5 mL centrifuge tube. Total RNA was eluted in 40 μL of Rnase-free H_2_O, and 1 μl RNase inhibitor was immediately added to each sample. One micro liter samples were taken and quantified using a Qubit^®^ 2.0 fluorometer (Invitrogen, United States). The extracted RNA was stored in a − 80°C refrigerator for later use.

### Library preparation

Depending on the RNA concentration, 3–4 groups of tick RNA with the equivalent mass of total RNA belonging to the same collection site were mixed to generate a total of 18 RNA library pools. Enrichment of RNA, then use NEBNext^®^ rRNA to remove kits rRNA. Following the manufacturer’s instructions, set the condition as follows: 94°C 7 Min, 75°C 2 Min, 70°C 2 Min, 65°C 2 Min, 60°C 2 Min, 55°C 2 Min, 37°C 5 Min, 25°C 5 Min, and hold at 4°C. Next, the NEBNext^®^ ultra II directional RNA library prep Kit for Illumina was used to construct the library according to the manufacturer’s instructions. First, the de-host library samples were fragmented, and then the first strand of cDNA and the second strand of cDNA were synthesized. The end of the next generation phosphorylation level, and dA short tail and the connection joint. To make the molecules attached to the adaptor available for subsequent PCR reactions, the USER^®^ enzyme was used to remove uracil from the NEBNext adaptor ring structure. Finally, PCR enrichment was carried out using the NEBNext^®^ multiplex oligos for Illumina (96 index primers) Kit, followed by RNA library purification.

### Next-generation sequencing and bioinformatics analysis

The 18 pools of completed tick have been sequenced in Illumina NovaSeq 6000, and the sequencing data in fastq.gz format were obtained by the automatic running of the sequencer. The remaining filtered sequences were then used to assemble viral fragments using SPAdes Genomics Workbench v3.13.0. The assembled contigs were compared to the non-redundant nucleotide (NT) and protein (NR) database using BLASTx and BLASTn with an E-value cut-off of <10^−5^ (Camacho et al., 2009; Buchfink et al., 2015). The highest BLAST hit for each contig was selected to identify contigs associated with viruses.

### Virus sequence confirmation and repair

For the viral sequences assembled, Premier V5.0 was used to design primers (primers are provided in Supplementary material S4) and subsequently RT-PCR was applied to verify which RNA sample contained the target sequence. Using the Hieff^®^ qPCR SYBR Green Master mix (Low Rox Plus) kit (Yeasen Biotechnology, China), the cycling program was set to pre-denaturing at 95°C for 5 Min, (40 cycles) denaturing at 95°C for 10s and finally annealing at 60°C for 30s. The positive viral sequences obtained by RT-PCR were mapped to the target sequence using CLC Genomics Workbench 12.0 to determine the sequencing depth and coverage (Misner et al., 2013). Next, primers were designed based on the depth sequencing results. The sequences obtained from the deep sequencing and assembly process were repaired using NEB Q5^®^ High-Fidelity 2X Master Mix. All PCR products were separated on a 1% agarose gel and positive PCR products were Sanger sequenced on an ABI 3730 DNA analyzer.

### Phylogenetic analyses

**Figure 2:**
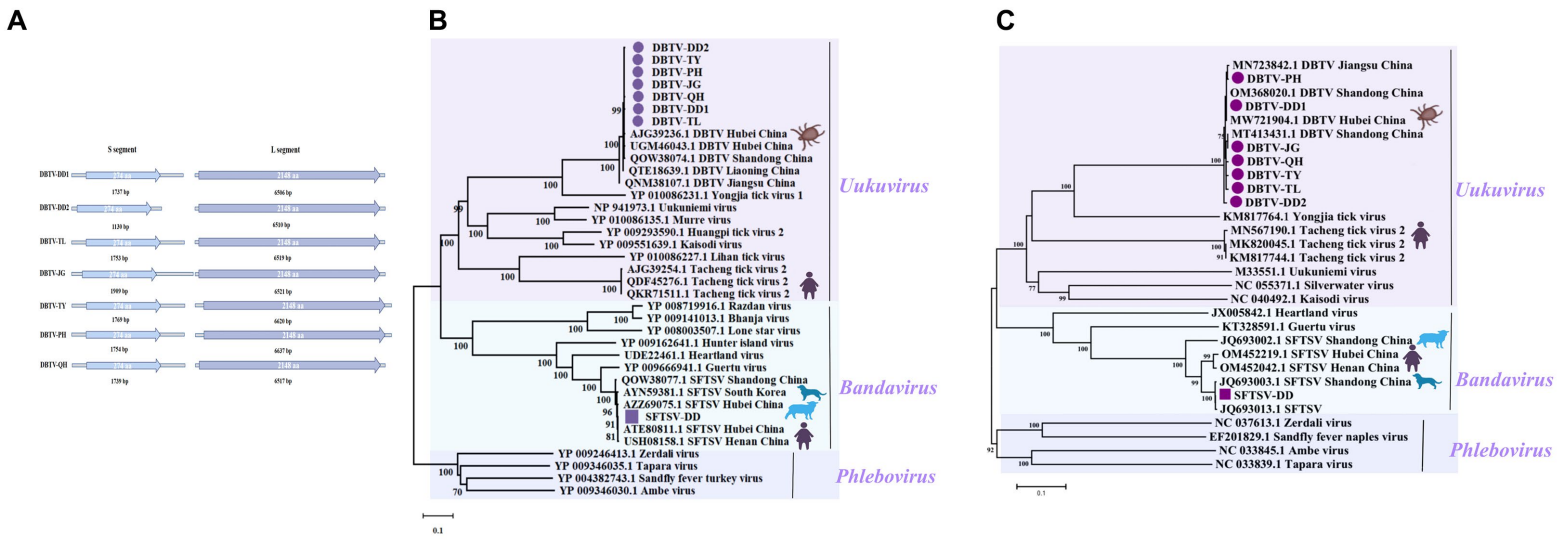
Genomic structure of the sequences of seven DBTVs and phylogenetic analysis of SFTSV-DD and DBTV. **(A)** The sequence structure of each DBTV, and the length of the encoded amino acids. **(B)** Phylogenetic trees of SFTSV-DD and 7 DBTVs based on RdRp sequence of members within *Phenuiviridae*. **(C)** Phylogenetic trees of SFTSV-DD and 7 DBTVs based on S segment of members within *Phenuiviridae*. Nodes with bootstrap values >70 are noted. Scale bars indicate substitutions per site. Animal icons represent host species. The circles marked in purple indicate the DBTV sequences obtained in this study. Those marked with purple squares depict SFTSV-DD sequences.

**Figure 3:**
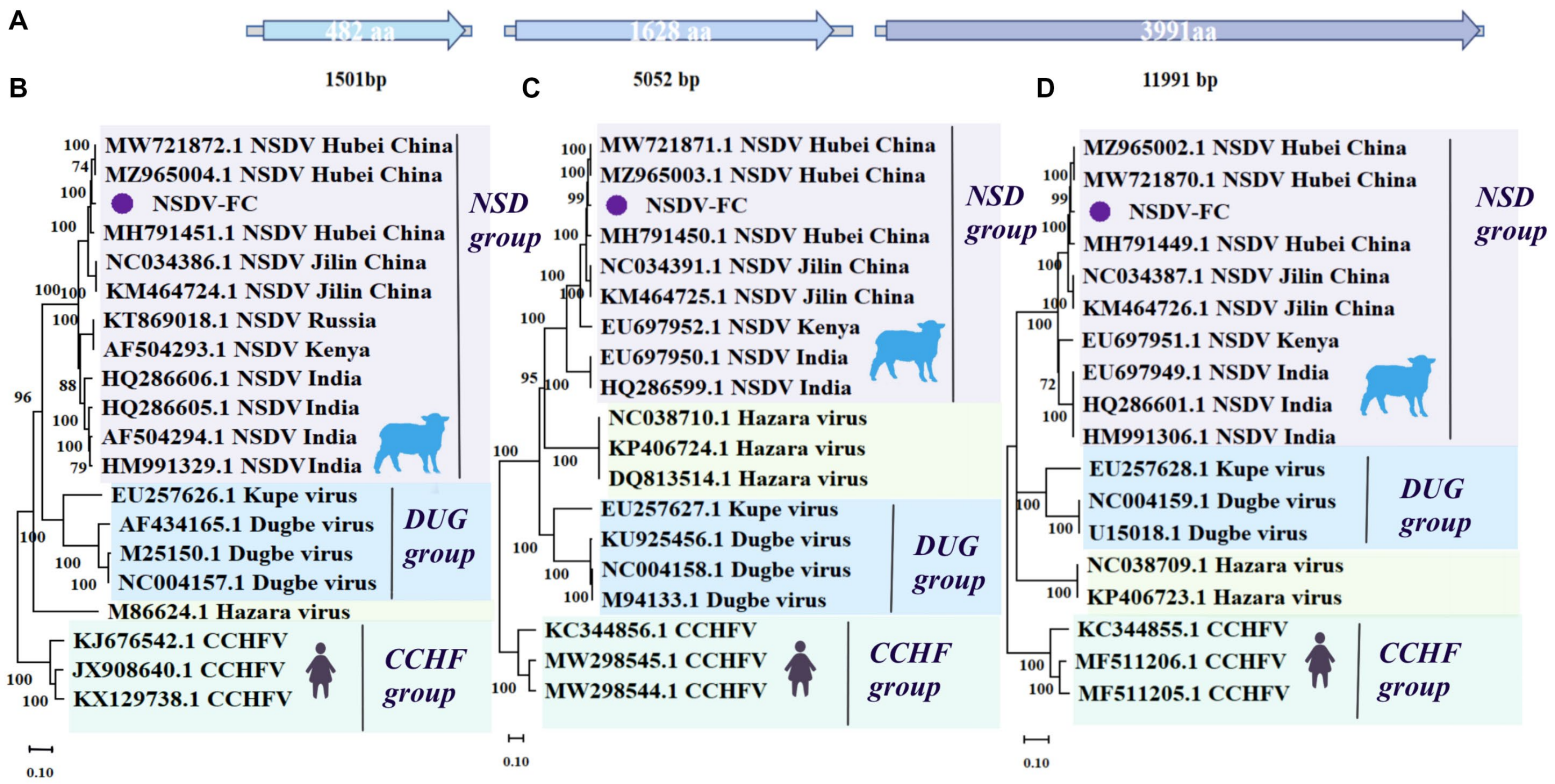
Genome structure and phylogenetic analysis of NSDV-FC. **(A)** Genomic structure of NSDV-FC. **(B)** Phylogenetic trees of NSDV-FC (1,501 bp) based on S segment of members within *Nairoviridae*. **(C)** Phylogenetic trees of NSDV-FC (5,052 bp) based on M segment of members within *Nairoviridae*. **(D)** Phylogenetic trees of NSDV-FC (11,991  bp) based on L segment of members within *Nairoviridae*. Nodes with bootstrap values >70 are noted. Scale bars indicate substitutions per site. Animal icons represent host species. Purple circles-marked strains were found in this research.

**Figure 4:**
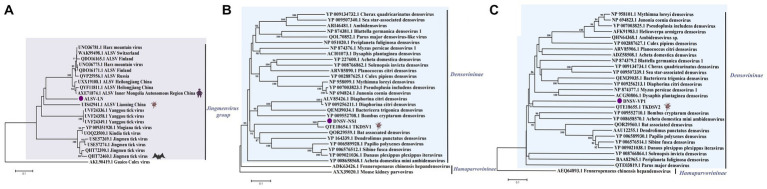
**(A)** Phylogenetic trees of ALSV-LN based on VP1a (121 aa) of members within *Jingmenvirus group*. **(B)** Phylogenetic trees of DNSV-NS1 (297 aa) based on NS1 of members within *Densovirinae*. **(C)** Phylogenetic trees of DNSV-VP1 (225 aa) based on VP1 of members within *Densovirinae*. Nodes with bootstrap values >70 are noted. Scale bars indicate substitutions per site. Animal icons represent host species. Purple circles-marked strains were found in this study.

**Figure 5:**
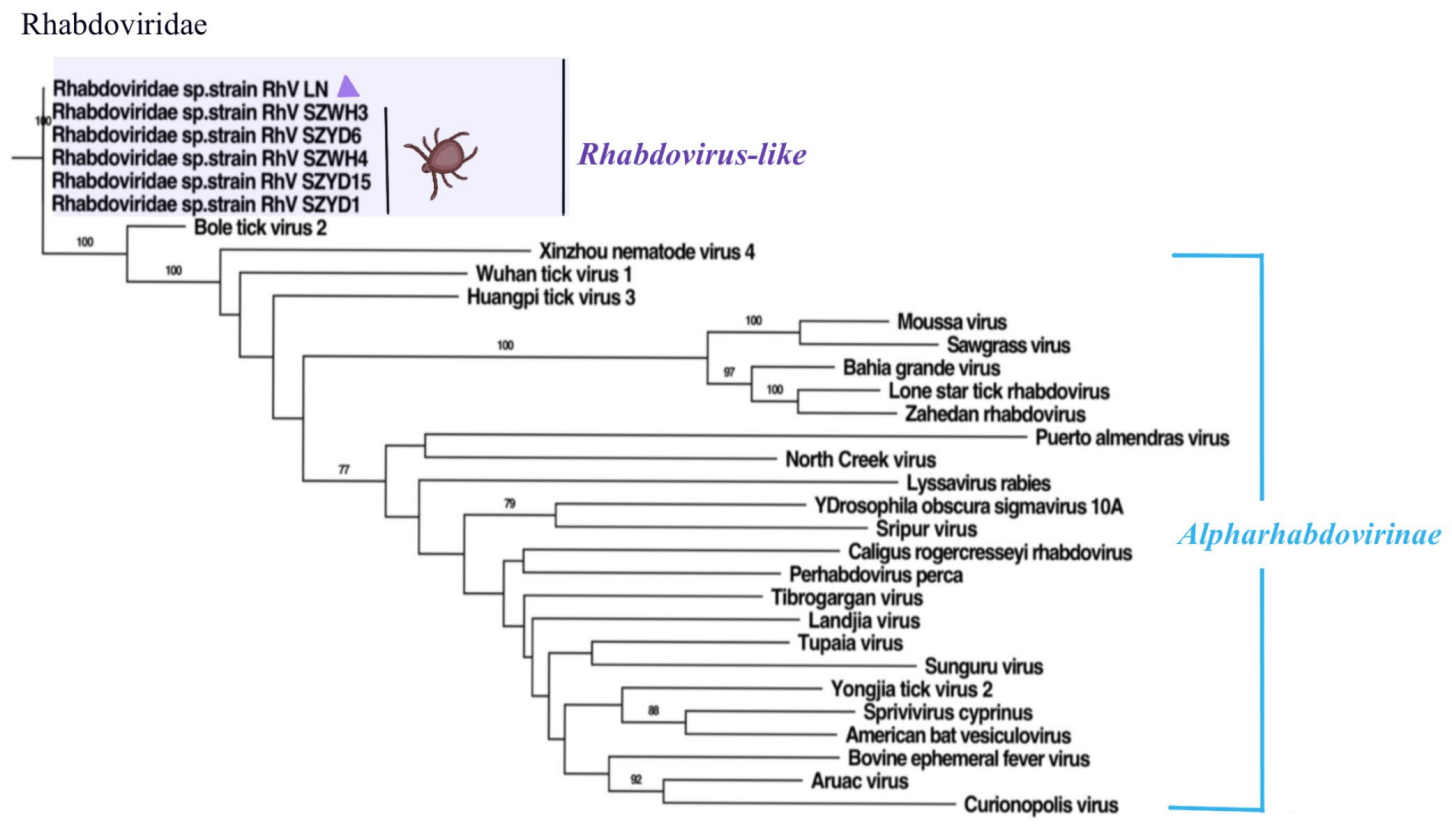
Phylogenetic trees of RhV/LN (340 aa) based on RdRp of members within *Rhabdoviridae*. Nodes with bootstrap values >70 are noted. Scale bars indicate substitutions per site. Animal icons represent host species. Purple triangles-marked strains were found in this research.

Based on the classification recommendations of The International Committee on Taxonomy of Viruses (ICTV)^1^ and the BLASTx and BLASTn results, for each potential virus, the viral nucleotide sequences or amino acid sequences obtained were compared with representative sequences of their corresponding virus families or genera for phylogenetic analysis. Sequences were aligned using MAFFT v7.450 (Katoh and Standley, 2013) or the ClustalW multiple sequence alignment program in MEGA version 11.0 (Kumar et al., 2018), and manually edited to remove any regions that were not aligned. Phylogenetic trees were then performed by the Neighbor-joining (NJ) method. Node support was determined by 1,000 bootstrap replicates in the NJ analysis (Figures 2–4). One of the phylogenetic analyses (Figure 5) was performed by using the best-fit alternative model generated by IQ-Tree 1.6.12 using the maximum likelihood method for each pair and the corresponding phylogenetic tree with 1,000 bootstrap replicates (Nguyen et al., 2015), finally implemented by Figtree V1.4.4 phylogenetic tree visualization.

## Results

### Viral metagenomics overview

**Figure 6:**
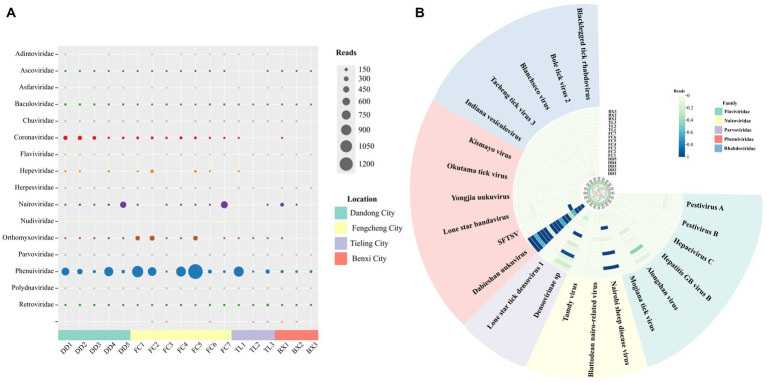
Viral presence and abundance across the pools. **(A)** The abundance of viral family reads in each pool. **(B)** The distribution of reads of tick-associated viruses in each pool.

A total of 561 ticks from the eastern part of Liaoning Province were collected in 2022 and divided into 58 nucleic acid pools. All ticks of the species *H. longicornis* were analyzed by morphological and molecular biological identification, and sequences from the sequencing results correlated with all COI barcode records in the NT database and the Barcode of Life Data System (BOLD). Eighteen library pools were generated for high-throughput sequencing, yielding a total of 709 million raw reads, with 318 million clean reads retained. The viral sequences were analyzed based on BLASTx and BLASTn, and contigs were aligned with NT or NR databases with E values ≤10^−5^ (Supplementary material S5). A large number of viral reads were annotated with information about 17 families of insects or mammal viruses (Figure 6A), such as *Flaviviridae*, *Nairoviridae*, *Phenuiviridae*, etc. Although the tick samples were sourced from the same border region of Liaoning Province, the virus reads differed between the pools. Based on sequencing data, this work focused on the distribution (Figure 6B) and characteristics of viruses in the tick-associated virus families *Flaviviridae*, *Nairoviridae*, *Parvoviridae*, *Phenuiviridae*, and *Rhabdoviridae*. Plant-hosted viruses and phages are widely distributed in metagenomic sequencing, but they do not have an excessive impact on biosafety and hence will not be discussed further in this research.

### Detection of viruses in ticks

The principal tick-associated viruses analyzed in this work were validated by RT-PCR (Supplementary materials S2, S3). Based on the results of high-throughput sequencing and RT-PCR, we investigated the minimum infection rate (MIR) of viruses with the whole genome (DBTV, SFTSV, NSDV), which were relatively widespread in the area of this collection than the other viruses (Table 1). Geographically, the minimum infection rate (MIR) of DBTV in ticks from Dandong city, Fengcheng city, Tieling city, and Benxi city, was 9.22% (19/206, 95% CI = 0.0579–0.1423), 6.67% (11/165, 95%CI = 0.0355–0.1192), 10% (10/100, 95%CI = 0.516–0.1804), 12.22% (11/90, 95%CI = 0.0655–0.2122), respectively (Table 1). The MIR of DBTV in this research was markedly higher than in previous surveys in the Shandong, Yunnan, and Guizhou provinces of China (Shao et al., 2020; Wang et al., 2021). This work presented positive for Severe fever with thrombocytopenia syndrome virus (SFTSV) in only four tick nucleic acid pools from Dandong City, with a total MIR of 0.71% (4/561, 95% CI = 0.0023–0.0194), which was compared to the MIR reported for SFTSV in Shandong Province which was slightly lower than previous studies (Shao et al., 2020). In addition, NSDV was detected in Dandong city, Fengcheng city, and Benxi city with a total MIR of 1.6% (4/561, 95% CI = 0.0078–0.0313) in this report.

**Table 1:** Positive rates for complete genome viruses collected in Liaoning Province, China.

Collection site	SFTSV		DBTV		NSDV	
	qRT-PCR	MIR %	qRT-PCR	MIR %	qRT-PCR	MIR %
Dandong city	4	1.94%	19	9.22%	4	1.94%
Fengcheng city	/	/	11	6.67%	1	0.61%
Tieling city	/	/	10	10%	/	/
Benxi city	/	/	11	12.22%	4	4.40%
Total	4	0.71%	51	9.09%	9	1.60%

### Phenuiviridae

SFTSV belongs to the *Bandavirus* genus, an emerging negative RNA bunyavirus first identified in 2009 in Henan Province, China, with clinical manifestations of severe fever, hemorrhage, leukocytopenia, and thrombocytopenia. The initial mortality rate reached up to 30%. Subsequently, cases of SFTSV infection from other Asian countries such as Korea, North Korea, Japan, and Vietnam have been reported, indicating that this novel virus appears to be circulating throughout Asia and poses a global public health threat (Yu et al., 2011; Zhan et al., 2017; Li et al., 2021). In this work, the complete L segment, M segment, and S segment of SFTSV were assembled from pools of Dandong city, tentatively named SFTSV-DD. The L segment encodes an RNA-dependent RNA polymerase (RdRp). A phylogenetic tree based on RdRp and S segments indicated that SFTSV-DD clusters with previously reported sequences of SFTSV isolates from dogs, sheep, ticks, and human hosts (Cheng et al., 2019; Shao et al., 2020; Dai et al., 2022), and form an evolutionarily closely related group of sequences that have fallen together with *Bandavirus* Species to cluster in the *Phenuiviridae* (Figures 2B,C). These SFTSVs were distributed worldwide and carried a high risk of pathogenicity, warning of the need for increased surveillance of susceptible areas to prevent disease infection in humans.

*Uukuvirus* is another influential genus of the *Phenuiviridae*, and the seven viruses with complete L and S segments of the coding region have been assembled from the pools of Dandong city (DBTV-DD1, DBTV-DD2), Fengcheng city (DBTV-JG, DBTV-TY, DBTV-PH, DBTV-QH), and Tieling city (DBTV-TL), respectively (Figure 2A), tentatively designated as Dabieshan tick virus strains (DBTV). The seven DBTVs share about 99% nucleotide identity with each other, and their L, S segment sequences all show about 98% nucleotide identity with the DBTV originally found in *H. longicornis* from Hubei Province, China. The L, and S sequences all shared about 98% nucleotide identity with the DBTV originally found in Hubei Province, China, and the L sequence shared 99% nucleotide identity with the DBTV recently found in Liaoning Province (Li et al., 2015; Shao et al., 2020; Xu et al., 2021).

Most of the seven DBTV strains in this research clustered together and were closely related to DBTV strains found in Shandong and Hubei provinces of China, forming a monophyletic group closely related to the viruses in the *Uukuvirus* genus. They also grouped with the Yongjia tick virus, Uukuniemi virus, and Tacheng tick virus 2 (Figures 2B,C), implying that they may have a common ancestor. Interestingly, in the phylogenetic tree of RdRp encoded by the L segment (Figure 2B), the seven DBTV species evolved in relation to QTE18639.1 DBTV reported from the same province of Liaoning, China (Yang et al., 2021), and were not as closely related to DBTV from Hubei and Shandong provinces in central China. In addition, a group of Tacheng tick viruses 2 (TcTV2) formed a separate branch in the *Uukuvirus* genus, which was located after the DBTV branch. Among these, TcTV2 (QKR71511), also identified using metagenomic sequencing analysis in a patient with a history of tick bites in northwest China, caused symptoms such as headache, fever, and erythema (Dong et al., 2021), suggesting that DBTV is widely distributed throughout China and potentially at risk of infecting humans.

### Nairoviridae

The family *Nairoviridae* spreads not only among arthropods but also among other rodents such as bats and birds. Tick-borne CCHFV represents the most influential nairovirus in terms of public health. It occurs widely in Europe, Asia, and much of Africa and causes acute fever with severe hemorrhage in humans (Garrison et al., 2020; Serretiello et al., 2020). CCHFV was regarded as a threat to bioterrorism and a virulent infectious virus to be monitored worldwide, but it was not detected in this study. NSDV refers to another virus of veterinary relevance from the *Nairoviridae* family, which initially caused fatal acute hemorrhagic gastroenteritis in small ruminants (mainly sheep, and goats) in eastern Africa (Krasteva et al., 2020). NSDV and its variant Ganjam virus have since been recorded in Asia, and NSDV infections in humans can cause fever, headache, and vomiting (Sudeep et al., 2009; Yadav et al., 2011).

In this work, the complete L, M, and S genomic segments of NSDV-FC were assembled and spliced, mainly from Fengcheng city, with lengths of 11,991 (L), 5,052 (M), and 1501 (S) bp, respectively (Figure 3A) covering the entire open reading frame (ORF) of the genome. They displayed essentially the same length as strains previously identified in China, India, and Africa. NSDV from the present study shared 97.24, 96.6, and 96.94% nucleotide identity with NSDV (L segment MW721870.1, M segment MW721871.1, S segment MW721872.1) identified from *H. longicornis* ticks collected from Hubei Province, China, respectively (Xu et al., 2021). The NSDV possessed a high degree of diversity, with the L, M, and S segments of NSDV-FC sharing about 94–95% nucleotide identity with strains from Jilin Province, China. Further, the L segment of NSDV-FC was more closely related to the African and Indian viruses, with a nucleotide identity of around 93%. While the S, M segment shared about 89% nucleotide identity with the strains identified in Africa and India. We constructed a phylogenetic tree based on the L, M, and S segment sequences, showing that NSDV-FC formed a branch with a group of NSDV (Figures 3B–D). Notably, in terms of evolutionary relationships, NSDV-FC relates more closely to the NSDV of Hubei Province in central China than to the NSDV of Jilin Province, which is also part of northeastern China, among the three gene segments. According to previous reports, even though no serological evidence of NSDV infection in humans and animals has been documented in China, the virus has been isolated from multiple animals in Africa and India. And antibodies to NSDV have been detected in human and animal sera (Joshi et al., 2005; Sudeep et al., 2009; Krasteva et al., 2020). NSDV continues to be prevalent in *H. longicornis* ticks in northeastern and central China, and may potentially infect livestock in eastern Liaoning and northeastern China.

### Flaviviridae

The viruses in the family *Flaviviridae* spread widely around the world, mainly by arthropods (ticks, mosquitoes, etc.), and several important tick-borne viruses include Tick borne encephalitis virus (TBEV), West Nile virus (WNV), Japanese encephalitis virus (JEV). Also, in recent years, new unclassified segmented flavi-like viruses such as Jingmen tick virus, ALSV, Yanggou tick virus, and others have been discovered (Mukhopadhyay et al., 2005; Qin et al., 2014; Wang et al., 2019). *Flaviviridae* infections have caused varying degrees of illness or death in humans and animals, posing a serious threat to health and security worldwide.

In the study, a partial sequence of segment 2 of ALSV was assembled and tentatively named ALSV-LN. Based on BLAST analysis, ALSV-LN strain with about 80% nucleotide and amino acid identity to both Chinese and Russian isolates, and segment 2 encodes an upstream open reading frame (nuORF), the VP1a and VP1b proteins, and was specific for JMVs (Wang et al., 2019; Kholodilov et al., 2021). A phylogenetic analysis was performed based on the VP1a protein replicated in ALSV-LN segment 2 (Figure 4A). The results demonstrated that both strains ALSV-LN and ALSV identified from the same province of Liaoning, China, formed a separate small branch, probably due to regional proximity and similar evolutionary characteristics. They gathered in the same cluster lineage as ALSV identified in other Chinese provinces and cities, as well as in Russia. ALSV, an emerging tick-borne segmental flavivirus, potentially features a complex evolutionary history, and current data are uncertain as to the mechanism of recombination evolution. Our results reflect, in part, the local ecological adaptation of ALSV (e.g., northeastern China) and the regional nature of its evolution. Previous studies have shown ALSV to be highly pathogenic, suggesting that ALSV-LN may also replicate in cells and have the potential to infect humans or livestock (Wang et al., 2019; Kholodilov et al., 2020).

### Rhabdoviridae

This work identified a segment in the *Rhabdoviridae* family and designated it Rhabdoviridae sp. strain RhV/LN (RhV/LN). The phylogenetic analysis of RdRp revealed (Figure 5) that RhV/LN was in the unclassified Rhabdoviridae sp. branch, which contains all sequences identified in the *H. longicornis* ticks from Hubei Province, China, share 99.41% amino acid sequence identity (Xu et al., 2021). Notably, it was highly distinct from the other members of the family, expressing less than 60% amino acid identity. In addition, RhV/LN was the first to be identified in the eastern border region of Liaoning Province, highlighting the complexity of the tick-borne viral group and further suggesting a correlation between viral group composition and geographical location.

### Parvoviridae

In the *Parvoviridae* family, there exists the *Parvovirinae* subfamily infecting vertebrates as well as the *Densovirinae* subfamily infecting invertebrates. Two virus sequences related to the *Densovirinae* subfamily were spliced from Dandong city and Fengcheng city pools, temporarily named Densovirinae NS1 (DNSV-NS1) and Densovirinae VP1 (DNSV-VP1). At the amino acid level, DNSV-NS1 shared 73.52% amino acid identity with QTE18654.1TKDSV1, and DNSV-VP1 shared 91.47% amino acid identity with QTE18655.1TKDSV2 (Yang et al., 2021). The construction of a phylogenetic tree (Figures 4B,C) based on their replicated proteins shows that both form a single cluster together with the Densovirinae sp. strain identified from the same province with the same tick species. The viruses in the *Densovirinae* subfamily mostly infect invertebrate hosts. The criteria for the division of genera in the *Densovirinae* subfamily are that all viruses in a genus should be monophyletic and encode NS1 proteins with more than 30% identity to each other at the amino acid sequence level. In conclusion, we confirmed that DSNV belongs to the *Densovirinae* subfamily within the *Parvoviridae* family and that its specific genus and pathogenicity require further study.

## Discussion

Based on the results of this high-throughput sequencing, a total of 17 species infecting human viruses or potentially pathogenic families were detected in *H. longicornis* ticks. And the RNA virus sequences associated with phlebovirus, orthonairovirus, flavivirus, *Rhabdoviridae*, and *Densovirinae* were studied, with STFSV, NSDV, and ALSV being identified as high-risk viruses in this reseach. Notably, the MIR of DBTV in this study was significantly higher (9.09%) compared to previous tick DBTV surveys in Shandong, Yunnan, and Guizhou provinces. DBTV most likely predominates as a novel tick-borne virus in human populations. In addition, RhV/LN was reported for the first time in Liaoning Province, China, after being reported in Hubei Province, China. This research, therefore, not only enriches the diversity of tick-borne viruses but also reaffirms the need for continuous surveillance of tick-borne viruses in the border areas of northeastern China.

Despite the fact that tick samples originated from the same border region of Liaoning Province, virus reads varied among regions (Figures 6A,B), e.g., tick-borne viruses related to *Flaviviridae*, *Nairoviridae*, *Phenuiviridae*, and *Retroviridae* had reads in each library, while some tick-related virus families, such as *Adintoviridae*, *Hepeviridae*, had reads in only a minority of pools. The differences in the number of viruses detected compared to previous investigations of *H. longicornis* in forested areas of Liaoning Province seem to be due, on the one hand, to differences in the time and place of tick collection and, on the other hand, to the fact that some of the low concentrations of viruses were degraded under non-anthropogenic conditions (Yang et al., 2021). Additionally, sequencing methods and experimental reagents may contribute to differences in virus detection rates. The prevalence of viruses carried by ticks, therefore, varies slightly.

*Phenuiviridae* accounted for a relatively high proportion of reads across the libraries (Figure 6A) and SFTSV was detected in four of the tick nucleic acid pools, with a slightly lower MIR (0.71%) than previous findings from Shandong Province (Shao et al., 2020). In China, *H. longicornis* is a major vector of SFTSV, transmitted by both transovarial and transstadial modes to other mammals (Zhuang et al., 2018). SFTSV is also prevalent in domestic animals such as sheep, cattle, dogs, and pigs. As human range activities expand, the potential for infection from close contact between these animals and their owners, in addition, to tick bites, becomes apparent. Although, in this research, although parts of the border areas of northeastern China were surveyed, it is possible that the tick sampling sites were not large enough and the overall sample size was insufficient to fully understand the relationship between prevalence in each region. SFTSV and HRTV are both novel highly pathogenic TBVs that are prevalent in the United States and some East Asian countries with established links to serious human diseases (Shen et al., 2018). SFTSV poses an imminent public health threat, yet the mechanism of SFTSV infection remains unclear, no standard treatment protocol has been developed to prevent SFTSV infection, and there is no commercially available vaccine (Casel et al., 2021). Although it has been proposed that SFTSV could be transmitted by birds migrating to expand the range of the virus and that rearrangements and recombination may occur between SFTSV strains to facilitate virus evolution (Yun et al., 2015; Wu et al., 2021). However, in this survey, the L, M, and S segments of SFTSV- DD shared more than 97% identity with SFTSV sequences of patient and animal origin. Furthermore, SFTSV- DD tentatively did not form a unique evolutionary relationship with other SFTSVs in the phylogenetic tree (Figures 2B,C). Moreover, numerous studies have proven that SFTSV in genetic evolution was relatively stable and did not undergo rapid evolution in humans (Yun et al., 2020; Ikemori et al., 2021). However, with the spread of SFTSV across a variety of animals and between humans, it is hard to ensure that cross-species evolution does not occur. The results of the present data provide a positive contribution, to some extent, toward the study of SFTSV evolution in nature.

DBTV, another emerging tick-borne virus in the *Phenuiviridae*, was first detected in Hubei Province, China, in 2015 (Li et al., 2015). The virus subsequently continued to be detected in central and southern China, indicating the widespread presence of DBTV in China. In the present survey, DBTV with a MIR of 9.09% has been detected in 51 out of 58 groups, which is distinctly higher than previous surveys in Shandong, Yunnan, and Guizhou provinces of China. In comparison to their study, in this research, there may have been differences in the region where the samples were collected, the number of samples, and the methods used to pre-process the samples. In addition, the different kits used for nucleic acid extraction and the different sequencing platforms and methods used for sequencing resulted in a greater diversity of viruses. The RdRp phylogenetic analysis indicated that DBTV clustered closely with the Yongjia tick virus, Uukuniemi virus, and with a group of Tacheng tick viruses 2 (TcTV2) in the Uukuvirus genus, forming a separate branch (Figure 2B). Of these, TcTV2 is responsible for severe disease, and numerous studies have identified DBTV with such an evolutionary signature, further suggesting that DBTV may emerge as a human pathogen (Wang et al., 2021; Yang et al., 2021; He et al., 2022). Furthermore, in this research, the seven DBTV strains displayed a high degree of amino acid identity with each other, despite being the same tick species from different eastern border regions of Liaoning Province. Meanwhile, in the phylogenetic tree, they clustered together. We, therefore, speculate that DBTV may be relatively conserved in terms of evolution and host selection. It also emerged that two pools of *H. longicornis* ticks collected from Shandong Province, China, detected the co-occurrence of SFTSV and DBTV (Shao et al., 2020). A similar situation arose in our survey, where a similar co-infection of SFTSV and DBTV was exhibited in one of the pools, but the same problem of uncertainty as to whether the two pathogens derived from the same tick was also encountered. Therefore, in patients bitten by ticks, consideration should be given to whether co-infection is due to multiple viruses. However, limited genomic information is currently known about this novel DBTV virus, and DBTV and SFTSV have evolved into distinct phylogenetic lineages (Dai et al., 2022; Ma et al., 2022). Given the extensive distribution of DBTV, it is hard to ensure that DBTV will not become a severe pathogenic agent causing outbreaks in various provinces of China. There is an urgent need to study its pathogenesis in order to determine the prevalence and genetic characteristics of this virus as soon as possible.

The next part of our search concerns NSDV of the *Nairoviridae*, a lethal pathogen of small ruminants (e.g., sheep). Although no serological evidence of NSDV infection in humans and animals has been temporarily discovered in China, NSDV has been successfully isolated from various host animals (ticks, mosquitoes, sheep) and humans in Africa and South Asia. *Haemaphysalis intermedia* ticks have been considered as a possible vector for its transmission (Joshi et al., 2005; Sudeep et al., 2009; Krasteva et al., 2020). The virus was first detected among *H. longicornis* ticks from northeastern China in 2015 and subsequently also recorded in central China (Gong et al., 2015; Yang et al., 2019). In the present study, we similarly discovered NSDV in the same tick species, *H. longicornis*, in northeastern China and positively for NSDV in a group of nine ticks (9/58), while annotating the sequencing results as contigs for NSDV, with the sequences obtained covering the entire ORF of the genome. Phylogenetic analysis evidences the clustering of the NSDV identified for this work with previously studied strains from China, but also some sequence differences while reflecting the genetic diversity of the virus across countries. It is noteworthy that in the phylogenetic analysis, Chinese NSDV strain appears to be more closely related to the African strain than to the Indian strain in evolutionary terms. The results resembled those of Yang L et al. and suggested a potential evolutionary route for this virus (Yang et al., 2019). Indeed, their research showed that the common ancestor of NSDV in China and Africa probably originated in India, but not in terms of geographical distribution. Hence there exists a necessity to conduct a more extensive epidemiological investigation of NSDV in the Chinese region to determine whether it is an imported virus.

The *Flaviviridae* contains four main genera of viruses, of which the *Flavivirus* genus produces insect-borne viral encephalitis (e.g., TBEV, WNV, JEV) and systemic diseases such as hemorrhagic fever (e.g., dengue virus, yellow fever virus, and the newly discovered JMTV, ALSV), with no specific drugs or vaccines currently available for the diseases (Mukhopadhyay et al., 2005; Qin et al., 2014; Wang et al., 2019). In this study, a novel viral sequence associated with ALSV was identified. Although the full genome has not been obtained, we obtained a partial sequence of segment 2 of ALSV-LN, which shares 70.91% amino acid identity with strain AXE71874.1 ALSV, originally isolated from patient samples in northeastern China. The phylogenetic analysis indicated that ALSV-LN formed a separate minor branch in the Jingmen virus group (Figure 4A). According to ICTV recommendations, ALSV is an unclassified species in the genus *Flavivirus*, but it is in the segmented flavi-like group with Jingmen tick virus, Mogiana tick virus, Guaico Culex virus, Shuangao insect virus 7, etc. The delineation of new species in the genus *Flavivirus* requires consideration not only of nucleotide sequence relatedness and the resulting phylogeny but also of genetic relationships, host range, and other biological features. Many previous studies have examined the identification of ALSV in northeastern China, as well as in Russia and Finland, and the presence of the ALSV virus from northeastern China with *H. longicornis* tick species was also identified in this work, thereby further hinting that ALSV may be widely distributed among various tick species in the northeast. As the sequences we obtained differed considerably from those previously obtained, their pathogenicity requires additional study.

The *Rhabdoviridae* family is ecologically diverse, and its members infect plants and animals. Rhabdoviruses have also been detected in invertebrates (including arthropods), some of which may be unique hosts or may act as intermediate hosts for transmission to other animals or plants. The delineation of genera also requires significant differences in genome sequence and structure, antigenicity, and ecological characteristics (e.g., host range, pathobiology, and mode of transmission), as recommended by ICTV.^2^ Whereas RhV/LN has not been genome-wide and remains poorly studied, it seems likely that in the future, with more research on RhV/LN, they will be classified as a new genus of the *Rhabdoviridae* family.

Differences in environment and tick species may contribute to the diversity and abundance of viruses carried on ticks. *H. longicornis* is a common tick species in northeastern China with a wide geographic distribution and host diversity, transmitting at least 30 human pathogens and serving as a vector for a variety of pathogens of medical and veterinary significance (Zhao L. et al., 2020). In this report, ticks were collected for sequencing from environments along the border of Liaoning Province, but the proximity of the regions may not adequately reflect the differences in virome diversity and richness of tick species. For prevalence, the limitations of the tick sampling sites and animal samples in this study may not provide an adequate understanding of the prevalence and infection rates of tick-borne viruses in each region. Finally, although we analyzed the minimum infection rates of some tick-borne viruses in Liaoning Province, there is still a paucity of studies on the seroprevalence of these tick-borne viruses in humans, domestic animals, and wildlife.

The recurrence of tick-borne viruses (TBDs) and the frequent occurrence of novel TBD events in recent years have made the control and prevention of TBDs difficult. On the one hand, the abundance of forests in Liaoning and its mild and humid climate provides a favorable environment for ticks to reproduce and grow, and the frequent trade exchanges with Korea and Japan in Liaoning Province provide the basis for tick species diversity. On the other hand, it is mainly due to our limited knowledge of the background of tick-associated viral diseases and the complex mechanisms of their transmission in nature. To achieve these goals, firstly, virus surveillance should be strengthened in susceptible areas and regular testing in non-susceptible areas, and secondly, tick bites in outdoor areas such as jungle areas should be prevented by reducing skin exposure. Finally, given that human health relates to many aspects of the modern living environment, including the health of plants and animals, ecosystem stability, and natural climate change, future research should include modeling of infection and transmission and must adhere to a “one health” strategy (Ostfeld and Brunner, 2015; Carlson and Phelan, 2022).

## Conclusion

In conclusion, this study applied mNGS to systematically examine the virulence group carried by the dominant tick species *H. longicornis* in Liaoning Province, identifying the known viruses SFTSV and NSDV that cause disease in humans and animals, and our results also confirm the presence of rhabdo-, phlebo-, flavi-like, and parvovirus sequences in ticks. Not only does it validate the desirability of virus surveillance in areas where tick-borne diseases are endemic, but it also provides information for possible future outbreaks of infectious diseases. At the same time, it offers an excellent reference for assessing the risk of infection by tick bites in both humans and animals, as well as insight into the evolution of the virus concerning mechanisms of species transmission.

## Data availability statement

The data presented in the study are deposited in the Genbank and repository, accession number OQ716537-OQ716539, OQ716521-OQ716522, OQ716519-OQ716520, OQ716534-OQ716536, OQ716542, OQ716540, OQ716541.

## Author contributions

YB and YL designed the experiments and performed the experiments. YT and XH provided reagents and materials. YB, YL, JL, and FT analyzed the data. YB drafted the manuscript. All authors critically read and revised the manuscript.

## Funding

This research was supported by the National Key Research and Development Program of China (2020YFC2005405, 2020YFA0712100, 2020YFC0840805, and 2021YFC0863400).

## Conflict of interest

The authors declare that the research was conducted in the absence of any commercial or financial relationships that could be construed as a potential conflict of interest.

## Publisher’s note

All claims expressed in this article are solely those of the authors and do not necessarily represent those of their affiliated organizations, or those of the publisher, the editors and the reviewers. Any product that may be evaluated in this article, or claim that may be made by its manufacturer, is not guaranteed or endorsed by the publisher.

## Footnotes

^1^ https://talk.ictvonline.org/ictv-reports/ictv_online_report/

^2^ https://talk.ictvonline.org/ictv-reports/ictv_online_report/
